# Improved left atrial imaging in atrial fibrillation patients using novel ECG-gated vs. conventional non-gated cardiac MRA

**DOI:** 10.1186/1532-429X-15-S1-O50

**Published:** 2013-01-30

**Authors:** Douglas Sheffer, Eugene Kholmovski, Lowell Chang, Krishna N Velagapudi, Kavitha Damal, Nassir F Marrouche, Christopher McGann

**Affiliations:** 1 Department of Cardiology, University of Utah Health Sciences Center, Salt Lake City, UT, USA; 2 Comprehensive Arrhythmia Research and Management (CARMA) Center, University of Utah Health Sciences Center, Salt Lake City, UT, USA

## Background

In patients undergoing atrial fibrillation (AF) procedures, imaging of the left atrium (LA) and pulmonary veins (PV) is important for pre-ablation planning and to identify post ablation complications. Conventional MRA protocols use first-pass, non-gated sequences that require long breath-holds. Quality of non-gated MRA's can be challenging in sick or sedated patients. We developed a novel ECG-gated, respiratory navigated MRA sequence less dependent on patient compliance, which yields better clarity of LA anatomy.

## Methods

Eighty patients with AF underwent either conventional non-gated (n=40) vs. novel ECG-gated (n=40) MRA on a 3T Verio scanner (Siemens, Erlangen, Germany). All MRA's were performed using 0.1 mmol/kg Multihance (Bracco Diagnostics Inc., Princeton, NJ). A novel ECG-gated, respiratory navigated MRA was developed using 3D saturation recovery prepared, GRE sequence with fast contrast injection (half dose, 1.0 mL/sec) followed by slow infusion (half dose, 0.1 mL/sec). Saturation pulse was applied every heart beat and fat saturation was applied immediately before data acquisition during LA diastole. Additional scan parameters were: axial imaging volume, FOV =400x400x110, voxel size=1.25x1.25x2.5 mm, TR/TE=2.8/1.3ms, flip angle=15 degrees, TI=250ms, phase encoding direction: left to right. Typical scan time was 3-5 minutes.

Conventional non-gated MRA were performed with contrast injection rate 2.0 mL/sec and continuous data acquisition during single breath-hold (14 sec.). Scan parameters included: axial imaging volume, FOV=400x263x120 mm, voxel size=1.25x1.25x2.5, TR/TE=2.8/1.1ms, flip angle=27 degrees.

**Figure 1:**
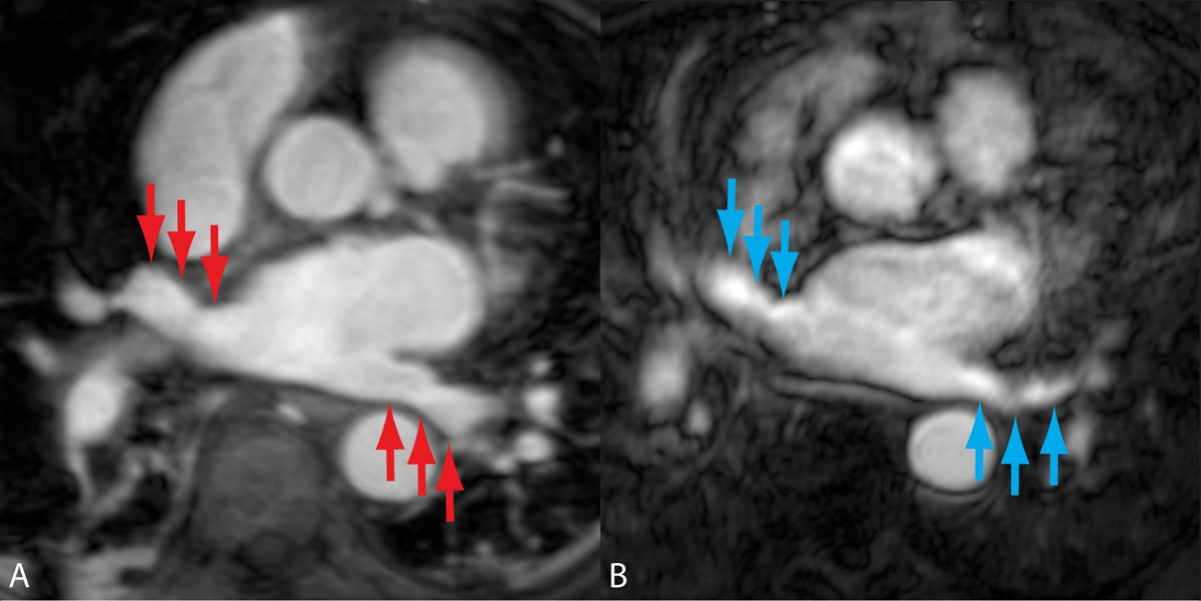
ECG-gated vs. non-gated cardiac MRA patient with atrial fibrillation. Nongated MRA (Image B) suffers from blurring and reduced border sharpness of the left atrial structures and pulmonary veins (blue arrows). In comparison, ECG-gated MRA (Image A) shows better border detail (red arrows) and overall scan quality without a significant loss in SNR and CNR. Images acquired in same atrial fibrillation patient, two days apart.

All MRA's were randomized and quality scores were determined by two experienced readers for contrast enhancement, border sharpness, and chamber detail of the PV's, LA, and LA appendage (LAA) (1=poor, 2=acceptable, 3=good, 4=excellent). (Figure 1)

## Results

ECG-gated MRA scored better than non-gated MRA in all categories. Quality of contrast for ECG-gated MRA averaged 3.18 vs. 2.63 quality score (QS; p-value < 0.0001) in the non-gated cohort. Border sharpness scored 3.08 vs. 2.35 QS (p-value < 0.0001) respectively. Chamber detail was also assessed with specific anatomical positions, which all yielded superiority of ECG-gated MRA (pulmonary veins 3.04 vs. 2.35 QS (p-value < 0.0001); left atrial appendage 3.11 vs. 2.25 QS (p-value < 0.0001); left atrium 3.08 vs. 2.35 QS (p-value < 0.0001).

## Conclusions

ECG-gated MRA improves image quality with better border sharpness and anatomical detail compared to conventional non-gated MRA. These advantages may translate into improved diagnostics in AF patients including detection of PV stenosis or following LA remodeling post-ablation.

## Funding

No disclosures.

